# Glutamate levels in the anterior cingulate cortex in un-medicated first episode psychosis: a proton magnetic resonance spectroscopy study

**DOI:** 10.1038/s41598-019-45018-0

**Published:** 2019-07-02

**Authors:** Faith R. Borgan, Sameer Jauhar, Robert A. McCutcheon, Fiona S. Pepper, Maria Rogdaki, David J. Lythgoe, Oliver D. Howes

**Affiliations:** 1 Psychosis Studies Department, Institute of Psychiatry, Psychology and Neuroscience, King’s College London, London, England; 2 Institute of Clinical Sciences, Faculty of Medicine, Imperial College London, Hammersmith Hospital, London, W12 0NN UK; 3 Centre for Neuroimaging Sciences, Institute of Psychiatry, Psychology and Neuroscience, King’s College London, London, England

**Keywords:** Cognitive neuroscience, Neuroscience

## Abstract

Converging lines of evidence suggest that glutamatergic dysfunction may contribute to the pathophysiology of first episode psychosis. We investigated whether first episode psychosis patients free from all pharmacological treatments and illicit substances show cortical glutamatergic alterations. One-hundred and eleven volunteers including 65 healthy volunteers and 46 first episode psychosis patients free from all pharmacological treatments (28 drug naïve) underwent a proton magnetic resonance spectroscopy scan measuring glutamate levels in the bilateral anterior cingulate cortex. Symptom severity was measured using the Positive and Negative Syndrome Scale (PANSS) and cognition was measured using the Wechsler Adult Intelligence Scale (WAIS) digit symbol test. There were no differences in glutamate levels between patients and controls. These findings remained unchanged when adjusting for the effects of age, sex and ethnicity or when restricting the analyses to patients who were both medication naïve to all pharmacological treatments and illicit substances. Whilst these findings do not preclude glutamatergic alterations in psychosis, methodological advances are needed for us to investigate whether patients show alterations in other aspects of glutamate function, such as pre-synaptic glutamate or release.

## Introduction

Schizophrenia and other psychotic disorders have a lifetime prevalence of approximately 0.7%^1,2^ and are ranked amongst the most disabling health conditions^3^, with annual costs ranging from 94 million to 102 billion dollars for each country, worldwide^4^. Although pharmacological treatments blocking the D2 dopamine receptor have been shown to reduce psychotic symptoms^5^, they are ineffective for approximately 30% of patients^6^ and they fail to improve cognitive impairments in the illness^7^. In view of these limitations, further work is needed to identify therapeutic mechanisms and biomarkers that may be useful for targeting both psychotic and cognitive symptoms.

Glutamate, the primary excitatory neurotransmitter in the central nervous system^8^, binds to a number of receptors in the brain including the N-methyl-D-aspartate receptor (NMDA), α-amino-3-hydroxy-5-metyl-4-isoxazolepropionic acid (AMPA) and kainate recepotors^8^. Converging lines of evidence suggest that glutamatergic dysfunction may contribute to the pathophysiology of psychosis^9,10^.

Glutamate theories of schizophrenia were developed following observations that N-methyl-D-aspartate receptor (NMDAR) antagonists induce positive and negative symptoms and cognitive impairments in healthy volunteers, paralleling symptoms seen in schizophrenia^11–13^. In particular, low doses of ketamine, an NMDAR antagonist, has been shown to induce psychotic symptoms, cognitive impairments and increase in glutamate levels in the anterior cingulate cortex in healthy volunteers^14^. Since ketamine has also been shown to exacerbate positive symptoms and cognitive impairments in schizophrenia^15,16^, it has been hypothesized that psychosis may be characterised by elevations in cortical glutamate^14^. However, other studies using comparable doses of ketamine have found that ketamine induces psychotic symptoms in healthy volunteers in the absence of any changes in glutamate levels in the anterior cingulate cortex^17,18^.

Glutamate levels have been investigated *in vivo* in psychosis using proton magnetic resonance spectroscopy (^1^H-MRS). However, discrepant findings have been reported. While decreased GLX (glutamate + glutamine) levels have been reported in the medial prefrontal cortex in 16 antipsychotic-naïve patients with first episode psychosis^19^, other studies have failed to find evidence of glutamate or GLX alterations in the anterior cingulate cortex in antipyshcotic-free patients with first episode psychosis^20–22^. Discrepant findings have also been reported in patients with chronic schizophrenia taking antipsychotics, with reports of no differences in GLX in grey matter^23^ but decreased glutamate levels in the left^24^ and bilateral anterior cingulate^25^. Meta-analyses have also come to discrepant conclusions, reporting no differences in glutamate levels in the medial frontal cortex in patients with first episode psychosis or chronic schizophrenia^26^ as well as decreased glutamate levels in the medial prefrontal cortex when combining patients with first episode psychosis and chronic schizophrenia^27^.

Several factors may account for these discrepant findings including illness chronicity and medication use. Although previous literature has shown that glutamate levels may alter during the course of illness^27^ and with the use of antipsychotic medication^28^, few studies^19–22^ have been conducted in antipsychotic free first episode psychosis patients. The largest study conducted to date, investigated glutamate levels in 45 antipsychotic free and 26 minimally treated patients in a multi-centre study in a multi-centre study^21^. However, this study included subjects taking compounds shown to influence glutamate levels, including illicit substances (cannabis^29^ and cocaine^30^) and concurrent treatments (benzodiazepines^31^ and antidepressants^32^). Moreover, this study did not to control for the potential confound of using three different scanners.

While smaller studies have also been conducted, these studies have used relatively small samples sizes (10–24 subjects per group)^19,20,22^ which, whilst well-powered to detect large effect sizes (*d* > *0*.*9*), lack statistical power to detect the moderate effect sizes (*d* = *0*.*4*–*0*.*6)* commonly seen in studies of psychotic disorders^33^. Moreover, many of these studies did not exclude spectra with poor signal-to-noise ratios or metabolite estimates with high variability; and they failed to use water suppression or cerebrospinal fluid contamination corrections, which can lead to artefacts and problems with spectral fitting^34^; and they also included some patients currently taking benzodiazepines^21,24^, shown to influence glutamate levels^31,35^.

In view of these methodological limitations and that no studies to date have investigated glutamate levels in patients with un-medicated first episode psychosis whilst controlling for the potential confounds of illicit substance use^29,30^ or concurrent pharmacological treatments that influence glutamate levels^31,32^, we aimed to investigate glutamate levels in drug-free first episode psychosis patients within three years of illness onset, excluding patients using illicit substances as well as other concurrent pharmacological treatments. In line with the evidence discussed above, we hypothesized that glutamate levels would be greater in un-medicated patients with first episode psychosis relative to healthy volunteers. We also hypothesized that greater glutamate levels in the anterior cingulate cortex would be associated with greater levels of symptom severity and poorer cognitive functioning.

## Results

### Demographics

There were no significant differences in age, sex, ethnicity between patients and controls. However, patients relative to controls had fewer years of education following compulsory education (see Table 1). Relative to healthy volunteers, patients performed significantly worse on the digit symbol coding test.

**Table 1:** Demographic, clinical and neurochemical measures.

	Healthy volunteers	First episode psychosis	t/x^2^	df	p
N	65	46	NA	NA	NA
Age mean (sd)	25.85 (4.87)	26.18 (4.42)	t = −0.357	102	0.722
Sex (male/female)	47/18	38/8	x^2^ = 1.708	1	0.191
Ethnicity (White Caucassian/Black African or Caribbean/Asian/mixed/missing data)	24/9/12/3/17	17/11/11/0/7	x^2^ = 0.819	1	0.365
Diagnosis (Schizophrenia/Schizoaffective disorder/Bipolar Disorder/Psychotic Depression)	NA	38/2/4/2	NA	NA	NA
Global Assessment of Functioning score mean (sd)	94.10 (10.29)	47.71 (17.72)	t = 10.901	48	<0.001
WAIS digit symbol coding test score mean (sd)	10.41 (3.02)	8.73 (2.62)	t = 2.263	58	0.027
PANSS positive mean (sd)	NA	14.33 (7.54)	NA	NA	NA
PANSS negative mean (sd)	NA	14.51 (8.46)	NA	NA	NA
PANSS general mean (sd)	NA	45.00 (23.41)	NA	NA	NA
PANSS total mean (sd)	NA	52.13 (24.50)	NA	NA	NA
Years of education after compulsory education*, mean (sd)	4.36 (1.91)	3.17 (2.42)	t = 2.389	74	0.019
Current antipsychotic use (yes/no)	0/65	0/46	NA	NA	NA
Current benzodiazepine use (yes/no)	0/65	0/46	NA	NA	NA
Current antidepressant use (yes/no)	0/65	0/46	NA	NA	NA
Medication naïve (yes/no)	NA	26/20	NA	NA	NA

*Years of compulsory education in the United Kingdom is age 16; NA = not applicable; SD = standard deviation. Group differences between patients and controls in demographic variables (age, sex, ethnicity) and clinical characteristics of the patient group.

### Metabolite levels in healthy volunteers acquired on scanner 1 and 2

Paired sample t-tests indicated that there were no statistically significant differences in glutamate estimates acquired on scanner 1 (M = 9.6; SD = 1.5) vs. scanner 2 (M = 8.3; SD = 0.9) (t(4) = 2.032, p = 0.112). An intra-class correlation reliability analysis indicated that there was good between-scanner reliability, α = 0.642. See Supplementary Materials for scanner differences in other metabolites (glutamine, GLX, NAA), spectra linewidth and signal to noise ratios.

### Glutamate levels in healthy volunteers and patients wtih first episode psychosis

**Figure 1:**
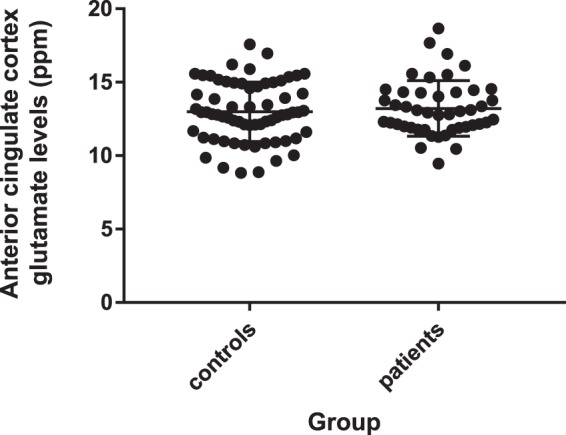
Anterior cingulate glutamate levels in first episode psychosis and healthy volunteers. Plot showing no significant differences in anterior cingulate cortex glutamate levels (ppm) between un-medicated patients with first episode psychosis and healthy volunteers (F(1,109) = 0.17, p = 0.68). Group means and standard deviations are shown.

Datasets acquired on scanner 1 and 2 showed a normal pattern of distribution as determined the Kolmogorov-Smirnov test. There were no significant differences between patients and controls in linewidth or signal to noise (see Supplementary Table 1). One healthy volunteer dataset that had glutamate levels exceeding the Cramér-Rao lower bounds ratio was excluded. There was no main effect of group on glutamate levels when adjusting for the effects of scanner (F(1,109) = 0.17, p = 0.68), (see Fig. 1), and this remained the case after adjusting for the effects of scanner, age, sex and ethnicity (F(1,83) = 0.15, p = 0.70). There was also no main effect of group on glutamate levels when adjusting for the effects of scanner and restricting the analysis to patients who were both medication naïve to all pharmacological treatments and free from all illicit substances (F(1,70) = 0.60, p = 0.44) or when restricting the analysis to patients diagnosed with schizophrenia who were both medication naïve to all pharmacological treatments and free from all illicit substances (F(1,66) = 0.61, p = 0.44). See Supplementary Materials for other metabolites. Since there were significant group differences in years of education following compulsory education, a post-hoc Pearson’s correlation coefficient was calculated to investigate the association between years of education and glutamate levels. There were no significant associations between years of education following compulsory education and glutamate levels in either controls (R = 0.05, p = 0.76) or patients (R = −0.15, p = 0.45).

### Relationship between metabolite levels and symptom severity

**Figure 2:**
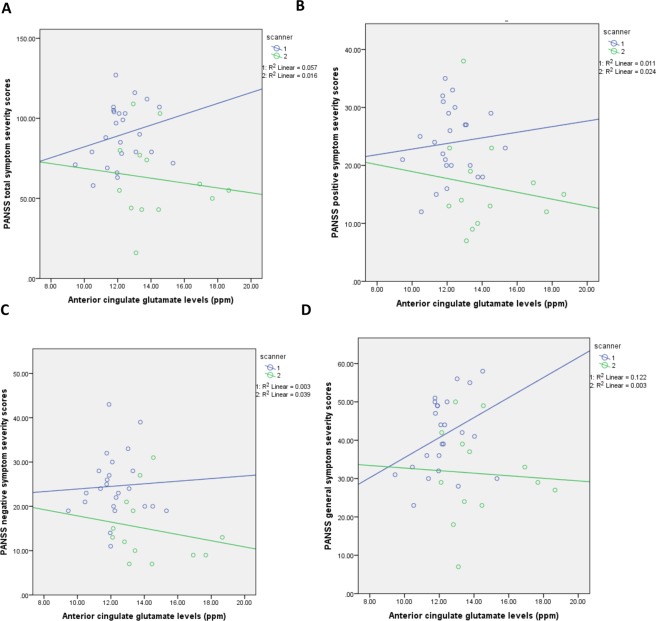
Association between anterior cingulate glutamate levels and symptom severity. Plot showing (**A**) no significant association between anterior cingulate glutamate levels (ppm) and PANSS total symptom severity scores for scanner 1 (ß = 3.39, SE = 2.95, p = 0.26, R^2^ = 0.06) or scanner 2 (ß = −3.10, SE = 2.47, p = 0.23, R^2^ = 0.09); (**B**) no significant association between anterior cingulate glutamate levels and PANSS positive symptom severity scores for scanner 1 (ß = 0.48, SE = 0.99, p = 0.63, R^2^ = 0.01) or scanner 2 (ß = −1.54, SE = 0.76, p = 0.06, R^2^ = 0.21); (**C**) no significant association between anterior cingulate glutamate levels and PANSS negative symptom severity scores for scanner 1 (ß = 0.29, SE = 1.18, p = 0.81, R^2^ = 0.003) or scanner 2 (ß = −0.13, SE = 0.76, p = 0.87, R^2^ = 0.002); (**D**) no significant association between anterior cingulate glutamate levels and PANSS general symptom severity scores for scanner 1 (ß = 2.61, SE = 1.49, p = 0.09, R^2^ = 0.09) or scanner 2 (ß = −0.85, SE = 1.20, p = 0.48, R^2^ = 0.02).

There was no significant association between glutamate levels and PANSS total (ß = −1.24, SE = 1.85, p = 0.51, R^2^ = 0.24), positive (ß = −0.96, SE = 0.59, p = 0.11, R^2^ = 0.22), negative (ß = −0.01, SE = 0.63, p = 0.99, R^2^ = 0.28) or general PANSS symptom severity scores (ß = 0.15, SE = 0.93, p = 0.87, R^2^ = 0.15), when adjusting for scanner. These findings remained unchanged when controlling for scanner differences, age, sex and ethnicity; and when restricting the analysis to patients who were both medication naïve to all pharmacological treatments and not currently using illicit substances; or when investigating these relationships separately for each scanner (see Fig. 2 and Supplementary Materials for results).

### Relationship between metabolite levels and cognition

Healthy volunteers showed no significant associations between glutamate levels and cognitive function, as determined by the digit symbol coding test (ß = −0.04, SE = 0.30, p = 0.90, R^2^ = 0.001). Similarly, patients also showed no significant associations between glutamate levels and cognitive function, as determined by the digit symbol coding test (ß = −0.16, SE = 0.29, p = 0.59, R^2^ = 0.01). These findings remained unchanged when controlling for scanner differences, age, sex and ethnicity; or when restricting the analysis to patients who were both medication naïve and not currently using illicit substances; or when investigating these relationships separately for each scanner (see Supplementary Materials).

## Discussion

Our main finding was that patients with un-medicated first episode psychosis do not show evidence of glutamate, glutamine or GLX abnormalities in the anterior cingulate cortex relative to healthy volunteers. These findings remained unchanged when restricting the analysis to patients diagnosed with schizophrenia who were medication naïve to all pharmacological treatments and not currently taking any illicit substances. We also showed that glutamate levels in the anterior cingulate were not linked to symptom severity or cognitive functioning.

Our findings are consistent with literature reporting that antipsychotic naïve patients with first episode psychosis show no differences in glutamate levels in the frontal cortex^20–22^ and meta-analytic findings showing no differences in glutamate levels in the frontal cortex in patients with first episode psychosis^26^. However, this study extends the largest study conducted in un-medicated patients with first episode psychosis^21^ in three key ways. Firstly, we controlled for the effects of scanner differences. Secondly, we excluded subjects taking compounds shown to influence glutamate levels including both illicit substances (e.g. cannabis^29^, cocaine^30^ and ketamine^14^) and concurrent pharmacological treatments known to influence glutamate levels (e.g. benzodiazepines^31^ and antidepressants^32^). Thirdly, by scaling metabolites to water as opposed to creatine^21^, we have avoided the potential confound of scaling metabolites to a reference compound that has been shown to be altered in schizophrenia^36^.

Although our findings are at odds with studies reporting decreased glutamate levels^19^ and greater glutamine levels^20^, several methodological differences may explain these discrepant findings. Firstly, these prior studies included patients currently taking benzodiazepines, shown to influence glutamate levels^31,35^. Secondly, these studies included spectra with poor signal-to-noise ratios and metabolites with high variability estimates ranging between 20–75%^20,24^. These methodological issues could be problematic because standard deviations greater than 20% introduce error and are typically indicative of poor data quality relating to artefacts arising from motion, frequency drift or poor spectral fitting. Moreover, it is unlikely that these studies were statistically powered to detect group differences in the context of such high variability since greater sample sizes are needed to detect group differences^37^. However, these discrepancies may also be linked to differences in scanner field strengths, voxel placements, imaging acquisition parameters and analytical techniques influencing spectral fitting (e.g. scaling metabolites to water vs. creatine, water suppression parameters and cerebrospinal fluid corrections) which may ultimately lead to a large degree in variability across studies. For example, if voxel placement methods differ, this will result in the measurement of different brain regions; if cerebrospinal correction methods differ, this will lead to the measurement of different tissue types (e.g. grey matter and cerebrospinal fluid vs. grey matter alone).

While we previously reported an inverse association between anterior cingulate cortex glutamate levels and positive symptom severity scores^38^, this study included some patients who had a history of exposure to pharmacological treatments and recreational illicit substances which, as discussed above, could have influence these findings. Our findings are also at odds with reports of a positive association between glutamate levels and total symptom severity scores^21^. The lack of consistency in the literature may be linked to a great deal of heterogeneity in proton magnetic spectroscopy measures owing to between-study heterogeneity in sample characteristics, imaging acquisition parameters and analytical techniques. The present findings therefore extend the existing literature to show that there is no association between anterior cingulate cortex glutamate levels and symptom severity scores in first episode psychosis patients diagnosed with schizophrenia who are both free from all pharmacological treatments and illicit substances, when controlling for the effects of age, sex and ethnicity.

Although a trend level association was observed between glutamate levels and positive symptom severity for data acquired using scanner 2, this relationship was not observed in the data acquired on scanner 1 or when combining both datasets. Moreover, although there was a trend level association between glutamate levels and general symptom severity scores for data acquired using scanner 1, this was not the case for data acquired on scanner 2 or when combining both datasets. These findings highlight the need for larger sample sizes to be used in order to avoid the risk of type II error^37^.

## Strengths and Limitations

A strength of the study was that no participants had been taking any compounds acting on the central nervous system, including pharmacological treatments or illicit substances. While we cannot exclude the possibility that the prior use of antipsychotics may have influenced the results, patients had a minimum drug washout period of 6 weeks and no patients had previously taken depot medications. Moreover, while we cannot exclude the possibility that prior illicit substance use may have influenced our findings, patients had negative urine drug screens prior to their scans on tests that able to detect cannabis, cocaine, amphetamine and opiate use and we excluded subjects with a history of substance use or dependence. Prior substance use is therefore unlikely to be a significant confound.

A methodological limitation of proton magnetic resonance spectroscopy is that it is not possible to distinguish between intra-cellular and extra-cellular glutamate levels or examine multiple brain regions simultaneously using the sequences that we used. Another limitation of this method is that it is not possible to correct for subject motion. This is a problem because scan to scan variations in phase and frequency of metabolite signals may result in incoherent averaging across frames, leading to reduced signal-to-noise^39^. A strength of the present investigation was that we used water suppression during imaging acquisition. Failure to use water suppression during imaging acquisition leads to a large water peak which may cause problems with spectral fitting^39^. While we cannot exclude the possibility that relaxation times may be different between patients and controls in the anterior cingulate cortex, future studies are needed to investigate this in order for us to identify if this needs to be taken into consideration in the analysis.

While the digit symbol coding test is highly correlated with global cognitive impairments, determined by comprehensive neuropsychological tests measuring attention, processing speed, executive function and working memory^40,41^, this measure lacks specificity at the expense of sensitivity and it is therefore unclear which precise aspect of cognition is affected. This measure was chosen to minimise subject burden, but, given our findings, it would be useful to determine if glutamate levels are linked to specific aspects of cognitive function in further studies. While we cannot exclude the possibility that our findings may have been influenced by group differences in years of education, this variable was not associated with glutamate levels in patients or controls and it is therefore unlikely to be a major confound.

A limitation of the study was that the data were acquired on two different scanners exhibiting different signal-to-noise ratios. Since we controlled for scanner differences in all analyses, a similar number of patients and controls were acquired on both scanners and we discarded spectra with poor signal-to-noise ratios, the use of different scanners is unlikely to influence the results. Moreover, our test-retest findings indicated that there were no significant differences between scanners for glutamate, glutamine or GLX estimates, supporting the integration these datasets.

Future studies using 7-Tesla scanners are needed to improve the quantification of metabolites and to improve signal-to-noise ratios. Previous literature has reported a 2.8-fold increase in signal-to-noise ratios and a significant decrease in the variability of metabolite estimates acquired on a 7-Tessla scanner relative to a 3-Tessla scanner^42^. These methodological advances will improve the validity and reliability of metabolite measurement and also enable us to estimate metabolites present at lower concentrations in the brain^42,43^. Preliminary studies using 7-Tesla scanners have shown that glutamate levels are lower in the dorsal anterior cingulate cortex in antipsychotic-treated patients with first episode psychosis relative to controls^44^ and that glutamate, glutamine and glutathione levels are lower in the anterior cingulate cortex in antipsychotic-treated patients with residual schizophrenia relative to controls^45^. However, future studies are needed to confirm this first episode psychosis without the confounds of medication or substance use.

## Implications

Glutamate metabolite estimates acquired using proton magnetic resonance spectroscopy reflect the average signal of intra-cellular and extra-cellular glutamate levels within a particular brain region^46^. In view of the limited spatial resolution of proton magnetic resonance spectroscopy, our finding that there were no significant  differences between patients and healthy volunteers does not exclude the possibility that there may be alterations in the cortical pre-synaptic synthesis of glutamate or glutamate release.

Since proton magnetic resonance spectroscopy provides the average signal of metabolites across all frames when subjects are at rest, motion artefacts that may influence metabolite fitting cannot be removed. In view of these limitations and that glutamatergic functioning is involved in the dynamic modulation of synaptic connections thought to underlie learning and memory^47,48^, our findings do not exclude the possibility that patients with first episode psychosis may show dynamic alterations in the glutamate system, involved in synaptic plasticity.

Our finding that patients and controls do not show differences in glutamate levels also does not exclude the possibility of NMDAR dysfunction in first episode psychosis. Future studies are needed to identify whether patients show NMDAR alterations and how such alterations might be linked to glutamatergic function. Since many of the patients included in the present investigation were medication naïve, it was not possible for us to stratify the sample according to treatment response. Since previous literature has shown that treatment resistant patients show greater glutamate levels relative to treatment responsive patients^49^, future longitudinal studies are needed to investigate the developmental trajectory of glutamatergic alterations in first episode psychosis, and how this relates to treatment response. It should also be noted that these findings do not preclude glutamatergic alterations in other brain regions^50–52^.

## Conclusions

We showed evidence for the first time, as far as we’re aware, that patients with first episode psychosis who are free from all pharmacological treatments and illicit substances, do not show glutamate alterations in the bilateral anterior cingulate cortex relative to healthy volunteers. Future studies are needed to investigate whether patients show glutamate alterations in other brain regions implicated in the pathophysiology of psychosis. Methodological advances are also needed to enable the investigation of the pre-synaptic synthesis of glutamate and glutamate release in schizophrenia.

## Methods

### Ethics statement

Ethical approvals were obtained from the local NHS Research Ethics Committees to conduct the study (Camberwell St. Giles, 14/LO/1289; Cambridge East, 12/EE/0220). The study adhered to the study protocol and guidelines described by the Declaration of Helsinki. Informed, written consent was obtained from all subjects.

### Subjects

A total of 111 volunteers (65 healthy volunteers, 46 un-medicated first episode psychosis patients) were recruited. All patients were recruited from the first episode psychosis services at South London Maudsley NHS Foundation Trust and the West London Mental Health NHS Trust. Age (age +/− 3 years) and sex-matched healthy volunteers were recruited via local advertising. Since two scanners were used for the study, an additional 5 healthy volunteers were recruited and scanned on two different scanners in order to determine inter-scanner variability. Data will be made available upon request.

### Inclusion/exclusion criteria

Volunteers were aged 18–35 and were able to give informed written consent. Volunteers were excluded if they had: 1) a history of a head injury leading to loss of consciousness, 2) personal or family history of neurological or physical health problems, 3) contraindications to MRI safety including head trauma, pregnancy, the presence of metal plates, pins, bridges or dentures; or 4) current history of substance use or dependence as determined by the Structured Clinical Interview for DSM-IV-TR (SCID-I/P)^53^.

First episode psychosis patients met following inclusion criteria: (1) diagnosis of a psychotic disorder as determined by the ICD-10; (2) antipsychotic naïve or antipsychotic free for at least 6 weeks, as determined by medical records and self-report; (3) no current use of any concurrent pharmacological treatments as determined by medical records and self-report (e.g. benzodiazepines, antidepressants etc.); (4) illness duration of less than three years as determined by medical records and self-report. Healthy volunteers met the additional inclusion criteria including: (1) no current or lifetime history of an Axis-I disorder as determined by the Structured Clinical Interview for DSM-IV-TR (SCID-I/P)^53^ and (2) no family history of an Axis-I disorder in first and second-degree relatives as determined by the Family Inventory for Genetics studies (FIGS)^54^.

### Demographics

Age, sex, ethnicity and years of education were recorded.

### Clinical measures

Clinical symptom severity was determined by trained staff using the Positive and Negative Syndrome scale (PANSS)^55^. Social and occupational functioning was measured using the Global Assessment of Functioning (GAF)^56^. Healthy volunteers were screened for personal and family history of mental health problems using SCID-I/P^53^ and FIGS^54^, respectively. Cognitive functioning was assessed using the Wechsler digit symbol coding test^57^. This was chosen based on previous findings that it is highly correlated with overall cognitive impairments in FEP as determined by comprehensive neuropsychological tests for attention, processing speed, executive function and working memory^40,41^.

### Neuroimaging

All 111 volunteers underwent an MRI scan at the Centre for Neuroimaging Sciences at King’s College London. Sixty-one scans (33 controls and 28 patients) were acquired using the General Electric MR750 3.0 T MRI scanner (scanner 1). Fifty scans (32 controls, 18 patients) were acquired on the General Electric (GE, Milwaukee, Wisconsin, USA) Signa HDxt 3.0 T MRI scanner (scanner 2). The dataset acquired on scanner 2 formed part of a larger study investigating the relationship between glutamate and dopamine^38^. While the aforementioned manuscript included 28 patients, only 17 of these patients met the inclusion criteria for the present study. An additional 5 healthy volunteers were scanned on both scanners to validate the integration of datasets acquired on different scanners.

### Neuroimaging acquisition

The neuroimaging acquisition parameters for scanner 1 and 2 were as follows: internal localizer scans were used to determine the anterior commissure-posterior commissure line and inter-hemispheric angle. For the voxel placements, 3D coronal inversion recovery prepared spoiled gradient echo (IR-SPGR) scans were acquired, followed by auto pre-scans for optimisation of water suppression and shimming. 1H-MRS spectra were acquired for the anterior cingulate region-of-interest (right-left 20 mm × anterior-posterior 20 mm × superior-inferior 20 mm). The placement of the anterior cingulate voxel was based on the midline sagittal localizer with the centre of the 20 mm × 20 mm × 20 mm voxel placed 13 mm above the anterior portion of the genu of the corpus callosum, perpendicular to the anterior commissure-posterior commissure line to minimize the inclusion of white matter and cerebral spinal fluid (CSF) (see Supplementary Fig. 1 for images of the coronal, axial and sagittal placement of the voxel). Finally, the ^1^H-MRS spectra (Point RESolves Spectroscopy (PRESS), TE = 30 ms, TR = 2 s) were obtained through the PROton Brain Examination (PROBE) sequence by GE, which includes water suppression.

### Neuroimaging analysis

The neuroimaging analysis methods for scanner 1 and 2 were as follows: water-scaled metabolites were analysed using LC-model 6.3-0I- using an experimentally acquired basis set to estimate concentrations of  16 metabolites including glutamate, glutamine, GLX (glutamate + glutamine), N-Acetyl-Aspartate (NAA), L-alanine, aspartate, creatine, phosphocreatine, GABA, glucose, glycerophosphocholine, glycine, myo-inositol, L-lactate, N-acetylaspartylglutamate, phosphocholine and taurine. We report metabolite values scaled to water, as opposed to creatine, based on previous literature indicating that creatine levels are lower in patients with schizophrenia relative to healthy volunteers^36^. Metabolite analyses were restricted to spectra with linewidth (full-width at half-maximum; FWHM) ≤ 0.1 ppm, Cramér-Rao lower bounds (CRLB) for glutamate ≤20%, signal to noise ratio ≥5. In-house scripts written in Python were used to identify the relative distribution of white matter, grey matter and cerebrospinal fluid in the 8 cm^3^ voxel prescribed to the anterior cingulate. The following correction was subsequently applied in order to correct for cerebrospinal fluid within the 20 mm^3^ voxel, where M = raw metabolite value, WM = white matter and GM = grey matter, as described previously^58^. In the equation, the numerator accounts for the fraction of each tissue type within the voxel, corrected by the water concentration in the tissue type. Since the initial LC model analysis was run assuming the voxel was pure white matter, the numerator is divided by the concentration of water in white matter. The denominator corrects for the assumption CSF doesn’t contain metabolites. No correction was applied for relaxation times, except for assuming the tissue water T2 = 80 ms. The equation also includes a correction for the default assumption the voxel is pure WM during the LCModel analysis.

$$Mcorr=M(\frac{WM+(GM\,\times 1.22)+(CSF\,\times 1.55)}{(WM+GM)})$$

### Statistical analyses

All statistical analyses were conducted using the Statistical Package for the Social Sciences (SPSS; Version 22). Normality of distribution was assessed using the Kolmogorov-Smirnov test. The significance threshold was p < 0.05 for all statistical tests and tests were two-tailed. Bonferroni corrections for multiple comparisons were used in cases where there was a statistically significant difference between groups, p < 0.05 (two-tailed).

Paired-samples t-tests were used to investigate differences in metabolite estimates, spectra linewidth, spectra standard deviations and signal to noise for 5 subjects who were scanned on both scanner 1 and 2. An intra-class correlation reliability analysis (ICC) was conducted in order to determine the reliability of metabolite levels estimates acquired on scanner 1 and 2.

A one-way analyses of covariance (ANCOVA) was conducted to determine whether there were differences in glutamate levels between patients and controls, when controlling for scanner differences. Secondary ANCOVAs were conducted to determine whether there were differences in other metabolite levels (glutamine, GLX and NAA) between patients and controls. Given the effects of age^59^, sex^25^ and ethnicity^60^ on metabolite levels, a one-way ANCOVA was also conducted to determine whether there were differences in metabolite levels between patients and controls when controlling for scanner differences, age, sex and ethnicity. Secondary ANCOVAs were also conducted to determine if there were differences in metabolite levels between patients and controls, adjusting for scanner differences, when restricting the analysis to 1) patients who were both medication naïve to all pharmacological treatments and free from all illicit substances; and when restricting the analysis to 2) patients satisfying the diagnostic criteria for schizophrenia who are both medication naïve to all pharmacological treatments and free from all illicit substances.

Multiple linear regressions were used to determine the relationship between glutamate levels and positive, negative, general and total symptom severity. A multiple linear regression model was used including glutamate levels and scanner as the independent variable and PANSS symptom severity scores as the dependent variable. Secondary multiple linear regressions were also conducted including scanner, age, sex and ethnicity as independent variables. Given the effects of antipsychotic medication^61^ and illicit substance use^29,30^ on metabolite levels, these analyses were repeated when restricting the analysis to patients who were medication naïve and also free from all illicit substances, adjusting for scanner. All of these analyses were repeated separately for datasets acquired on each scanner.

A multiple linear regression was used to determine the relationship between glutamate levels and cognitive function, as determined by the digit symbol coding test, separately for controls and patients. These linear regression models included glutamate and scanner as the independent variables and the cognitive tests as the dependent variable. Secondary multiple linear regressions were also conducted to determine the relationship between glutamate levels and cognitive function, when controlling for scanner differences, age, sex and ethnicity by including glutamate, scanner, age, sex and ethnicity as independent variables and the cognitive test scores as the dependent variable. Given the effects of antipsychotic medication^61^ and illicit substance use^29,30^ on metabolite levels, these analyses were repeated when restricting the analysis to patients who were medication naïve and also free from all illicit substances, adjusting for scanner. All of these analyses were also repeated separately for datasets acquired on each scanner. Since we observed group differences in years of education, following compulsory education, post-hoc Pearson’s correlation coefficients were calculated to investigate the association between glutamate levels and years of education, in each group.

## Supplementary information

Supplementary materials
