# Supplementary material for: Glutamate levels in the anterior cingulate cortex in un-medicated first episode psychosis: a proton magnetic resonance spectroscopy study
Source: Sci Rep. 2019 Jul 2;9:8685. doi: 10.1038/s41598-019-45018-0 (PMC6606579; doi:10.1038/s41598-019-45018-0)
Supplement: Supplementary file 1 — Supplementary materials [file 41598_2019_45018_MOESM1_ESM.pdf]

Supplementary material

Glutamate levels in the anterior cingulate cortex in un-medicated first episode psychosis: a proton magnetic resonance spectroscopy study

Faith R. Borgan, PhD [1,2]*, Sameer Jauhar, PhD[1*], Robert A. McCutcheon MRCPsych[1,2], Fiona S. Pepper, MSc[3], Maria Rogdaki MRCPsych[1,2], David J. Lythgoe, PhD[3], Oliver D. Howes, PhD[1,2]

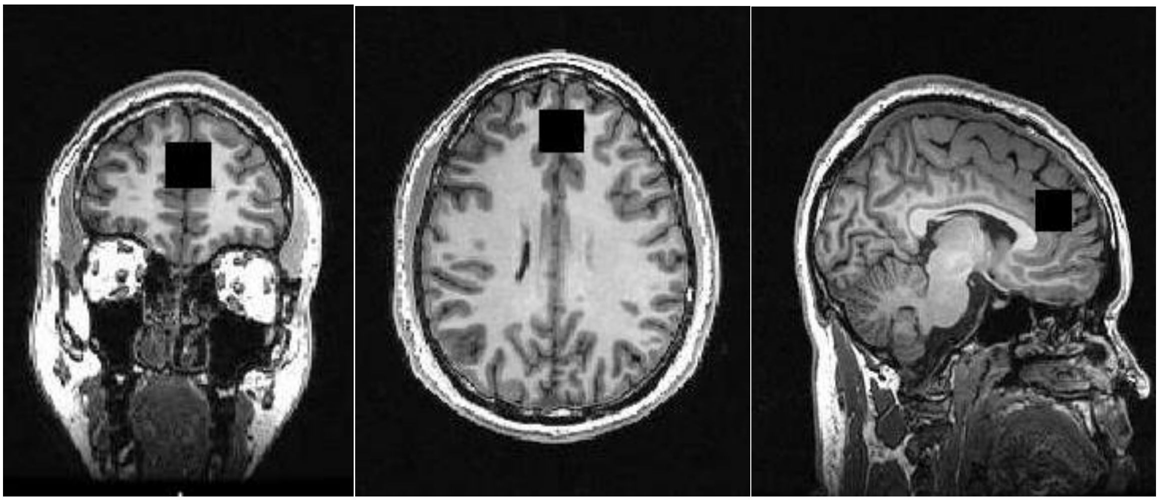

Supplementary figure 1. Coronal (left), axial (middle) and sagittal (right) planes depicting the placement of the voxel over the anterior cingulate during the proton magnetic resonance spectroscopy scan. The placement of the anterior cingulate voxel was based on the midline sagittal localizer with the centre of the 20 mm x 20 mm x 20 mm voxel placed 13 mm above the anterior portion of the genu of the corpus callosum, perpendicular to the anterior commissure-posterior commissure line to minimize the inclusion of white matter and cerebral spinal fluid.

Supplementary table 1. Proton magnetic resonance spectroscopy quality control data

	**Healthy volunteers**	**First episode psychosis**	**t**	**df**	**p**
N	65	46	NA	NA	NA
Glutamine mean (sd)	4.92 (2.55)	5.29 (2.80)	t=-0.729	109	0.468
GLX (glutamate + glutamine) mean (sd)	18.12 (3.65)	18.40 (3.82)	t=-0.403	109	0.688
N-Acetyl-Aspartate mean (sd)	12.33 (1.18)	12.10 (1.38)	t=1.088	109	0.279
Linewidth mean (sd)	0.04 (0.01)	0.037 (0.01)	t=-0.850	109	0.397
Signal to noise mean (sd)	23.17 (6.60)	24.10 (6.31)	t=-0.717	1-0	0.475

N=number; df=degrees of freedom; t=t statistic; sd=standard deviation

Metabolite levels in healthy volunteers acquired on scanner 1 and 2

Paired sample t-tests indicated that there were no statistically significant differences in 1) glutamate estimates acquired on scanner 1 (M=9.6; SD=1.5) vs. scanner 2 (M=8.3; SD=0.9), t(4)=2.032, p=0.11; 2); 2) glutamine estimates acquired on scanner 1 (M=3.4; SD=1.9) vs. scanner 2 (M=4.2; SD=0.8), t(4)=-1.19, p=0.30; 3) GLX estimates acquired on scanner 1 (M=13.1; SD=2.4) vs. scanner 2 (M=8.9; SD=0.7), t(4)=0.552, p=0.61. However,

a paired samples t-test indicated that NAA estimates were significantly higher on scanner 1 (M=8.9; SD=0.7) vs. scanner 2 (M=7.8; SD=0.7), t(4)=5.54, p=0.005. An intra-class correlation reliability analysis indicated good between-scanner reliability, α=0.642. However, a paired sample t-tests indicated that spectra linewidth was significantly higher for metabolites acquired on scanner 1 (M=0.6; SD=0.01) compared to scanner 2 (M=0.04; SD=0.1), t(134)=-2.45, p=0.02. A paired sample t-test indicated that there were no differences in the standard deviations from glutamate between scanner 1 (M=6.8; SD=0.44) or 2 (M=6.4; SD=1.14), t(4)=-1.00, p=0.37. A paired sample t-test indicated signal to noise was significantly higher on scanner 1 (M=26.1; SD=6.4) compared to scanner 2 (M=19.4; SD=4.7), t(134)=6.96, p<0.001. In view of these differences, we included scanner as a co-variate in all analyses.

Metabolite levels in healthy volunteers and first episode psychosis patients

Glutamine

Sixty glutamine datasets exceeding the Cramér-Rao lower bounds ratio was excluded. There was no main effect of group on glutamine levels when adjusting for scanner (F(1,50)=0.27, p=0.61), and this remained the case after adjusting for the effects of scanner, age, sex and ethnicity (F(1,33)=0.56, p=0.46). There was also no main effect of group on glutamine levels when adjusting for scanner and restricting the analysis to medication naïve patients who were free from illicit substances (F(1,26)=0.26, p=0.62) or when restricting the analysis to medication naïve patients, free from all illicit substances who also met the diagnostic criteria for schizophrenia (F(1,22)=0.33, p=0.57).

GLX

No GLX datasets exceeded the Cramér-Rao lower bounds ratio. There was no main effect of group on GLX levels (F(1,110)=1.92, p=0.17) and this remained the case after adjusting for the effects of scanner, age, sex and ethnicity (F(1, 84)=1.53, p=0.22). There was also no main effect of group on GLX levels when adjusting for the effects of scanner and restricting the analysis to medication naïve patients who were also free from illicit substances (F(1,71)=8.56, p=0.14) or when restricting the analysis to medication naïve patients, free from all illicit substances who also met the diagnostic criteria for schizophrenia (F(1,67)=1.45, p=0.23).

NAA

No NAA datasets exceeded the Cramér-Rao lower bounds ratio. There was no main effect of group on NAA levels (F(1,106)=0.95, p=0.33), and this remained the case after adjusting for the effects of scanner, age, sex and ethnicity (F(1, 84)=0.78, p=0.38). There was no main effect of group on NAA levels when adjusting for the effects of scanner and restricting the analysis to medication naïve patients who were also free from illicit substances (F(1,71)=1.16, p=0.29) or when restricting the analysis to medication naïve patients, free from all illicit substances who also met the diagnostic criteria for schizophrenia (F(1,67)=2.74, p=0.10).

Relationship between metabolite levels and symptom severity

Total symptom severity scores

There was no relationship between glutamate levels and PANSS total symptom severity scores when adjusting for scanner (ß=-1.24, SE=1.85, p=0.51, R^2=0.24) and this remained the case when restricting the analysis to patients who were both medication naïve and not taking illicit substances (ß=-0.98, SE=2.69, p=0.72, R^2=0.11). There was also no relationship between glutamate levels and PANSS total symptom severity scores when adjusting for scanner differences, age, sex and ethnicity (ß=-1.02, SE=2.12, p=0.63, R^2=0.22).

When investigating these relationships separately for each scanner, there was no relationship between glutamate levels and PANSS total symptom severity scores for scanner 1 (ß=3.39, SE=2.95, p=0.26, R^2=0.06) or scanner 2 (ß=-3.10, SE=2.47, p=0.23, R^2=0.09) (see figure 2A), and this remained the case when adjusting for the effects of age, sex and ethnicity for scanner 1 (ß=7.09, SE=3.96, p=0.09, R^2=0.21) and scanner 2 (ß=-2.86, SE=2.74, p=0.32, R^2=0.28). These findings remain unchanged when restricting the analysis to patients who were both medication naïve and not currently using illicit substances for scanner 1 (ß=4.17, SE=3.68, p=0.28, R^2=0.08) and scanner 2 (ß=-6.02, SE=2.76, p=0.12, R^2=0.61).

Positive symptom severity scores

There was no relationship between glutamate levels and PANSS positive symptom severity scores (ß=-0.96, SE=0.59, p=0.11, R^2=0.22), and this remained the case when restricting the analysis to patients who were both medication naïve and not taking illicit substances (ß=-0.45, SE=0.97, p=0.65, R^2=0.07). There was also no

relationship between glutamate levels and PANSS positive symptom severity scores when adjusting for scanner differences, age, sex and ethnicity (ß=-0.83, SE=0.66, p=0.22, R^2=0.12).

There was also no relationship between glutamate levels and PANSS positive symptom severity scores for scanner 1 (ß=0.48, SE=0.99, p=0.63, R^2=0.01) or scanner 2 (ß=-1.54, SE=0.76, p=0.06, R^2=0.21) (figure 2B), and this remained the case when adjusting for the effects of age, sex and ethnicity for scanner 1 (ß=2.09, SE=1.32, p=0.13, R^2=0.20) and scanner 2 (ß=-1.23, SE=0.72, p=0.12, R^2=0.52). These findings remain unchanged when restricting the analysis to patients who were both medication naïve and not currently using illicit substances for scanner 1 (ß=0.95, SE=1.16, p=0.42, R^2=0.043) and scanner 2 (ß=-0.16, SE=1.10, p=0.89, R^2=0.002).

Negative symptom severity scores

There was no relationship between glutamate levels and PANSS negative symptom severity scores (ß=-0.01, SE=0.63, p=0.99, R^2=0.28) and this remained the case when restricting the analysis to patients who were both medication naïve and not taking illicit substances (ß=-0.52, SE=1.04, p=0.62, R^2=0.08). There was also no relationship between glutamate levels and PANSS negative symptom severity scores when adjusting for the effects of scanner, age, sex and ethnicity (ß=-0.17, SE=0.66, p=0.80, R^2=0.26).

Similarly, there was no relationship between glutamate levels and PANSS negative symptom severity scores for scanner 1 (ß=0.29, SE=1.18, p=0.81, R^2=0.003) or scanner 2 (ß=-.13, SE=0.76, p=0.87, R^2=0.002) (see figure 2C), and this remained the case when adjusting for the effects of age, sex and ethnicity for scanner 1 (ß=0.14, SE=1.53, p=0.93, R^2=0.20) and scanner 2 (ß=-0.17, SE=0.85, p=0.84, R^2=0.03). These findings remain unchanged when restricting the analysis to patients who were both medication naïve and not currently using illicit substances for scanner 1 (ß=0.44, SE=1.53, p=0.78, R^2=0.01) and scanner 2 (ß=-0.25, SE=0.76, p=0.75, R^2=-0.06).

General symptom severity scores

There was no relationship between glutamate levels and general symptom severity scores (ß=0.15, SE=0.93, p=0.87, R^2=0.15), and this remained the case when restricting the analysis to patients who were both medication naïve and not taking illicit substances (ß=-0.01, SE=1.36, p=0.99, R^2=0.03). There was also no

relationship between glutamate levels and PANSS general symptom severity scores when adjusting for scanner differences, age, sex and ethnicity (ß=0.45, SE=1.06, p=0.67, R^2=0.14).

There was also no relationship between glutamate levels and PANSS general symptom severity scores for scanner 1 (ß=2.61, SE=1.49, p=0.09, R^2=0.09) or scanner 2 (ß=-0.85, SE=1.20, p=0.48, R^2=0.02) (see figure 2D), and this remained the case when adjusting for the effects of age, sex and ethnicity for scanner 2 (ß=-0.89, SE=1.37, p=0.53, R^2=0.22) but not scanner 1 (ß=4.85, SE=1.88, p=0.02, R^2=0.34). There was no association between glutamate levels and PANSS general symptom severity scores or when restricting the analysis to patients who were both medication naïve and not currently using illicit substances for scanner 1 (ß=2.78, SE=1.88, p=0.16, R^2=0.13) and scanner 2 (ß=-0.08, SE=1.47, p=0.95, R^2=0.00).

Relationship between metabolite levels and cognition

Healthy volunteers showed no association between glutamate levels and cognitive function, as determined by the digit symbol coding test (ß=-0.04, SE=0.30, p=0.90, R^2=0.001, and this remained the case when controlling for scanner differences, age, sex and ethnicity (ß=-0.03, SE=.31, p=0.92, R^2=0.04) or when investigating this relationship separately for scanner 1 (ß=-0.24, SE=.47, p=0.61, R^2=0.01) and 2 (ß=0.71, SE=.55, p=0.22, R^2=0.13).

Patients also showed no association between glutamate levels and cognitive function, as determined by the digit symbol coding test (ß=-0.16, SE=0.29, p=0.60, R^2=0.01), and this remained the case when controlling for scanner differences, age, sex and ethnicity (ß=-.13, SE=0.32, p=0.69, R^2=0.06). There was no association between glutamate levels and the digit symbol coding test when restricting the analysis to patients who were both medication naïve and not currently using illicit substances for scanner 1 (ß=0.41, SE=0.51, p=0.44, R^2=0.07) and scanner 2 (ß=0.41, SE=0.51, p=0.44, R^2=0.07). There was no association between glutamate levels and the digit symbol coding test when restricting the analysis to patients who were both medication naïve and not currently using illicit substances for scanner 1 (ß=-0.07, SE=0.38, p=0.85, R^2=0.001) and scanner 2 (ß=0.42, SE=0.45, p=0.37, R^2=0.08).
